# CD8^+^ T cells are present at low levels in the white matter with physiological and pathological aging

**DOI:** 10.18632/aging.104043

**Published:** 2020-10-13

**Authors:** Manuel Moreno-Valladares, Tulio M. Silva, Juan P. Garcés, Ander Saenz-Antoñanzas, Leire Moreno-Cugnon, María Álvarez-Satta, Ander Matheu

**Affiliations:** 1 Biodonostia Health Research Institute, Group of Cellular Oncology, San Sebastian, Spain; 2 Donostia University Hospital, Pathology Department, San Sebastian, Spain; 3 CIBER of Frailty and Healthy Aging (CIBERfes), Carlos III Institute, Madrid, Spain; 4 IKERBASQUE, Basque Foundation for Science, Bilbao, Spain

**Keywords:** aging, neuroscience, pathology

## Abstract

The presence and functional role of T cell infiltration in human brain parenchyma with normal aging and neurodegeneration is still under intense debate. Recently, CD8^+^ cells have been shown to infiltrate the subventricular zone in humans and mice with a deleterious effect on neural stem cells. However, to which extent T cell infiltration in humans also occurs in other regions such as cortical areas and, especially, white matter (WM) has not yet been addressed. In this work, we report a low-grade infiltration of T cells (CD3^+^, CD4^+^ and CD8^+^) in the WM of aged individuals that is also observed at similar levels in patients with neurodegenerative disorders (Alzheimer´s disease). In particular, CD3^+^ and CD8^+^ cells were increased in perivascular and parenchymal WM and cortical regions (enthorinal cortex). These results reveal that T cell infiltration in brain parenchyma occurs with physiological and pathological aging in several regions, but it seems to be lower than in the subventricular zone neurogenic niche.

## INTRODUCTION

Aging consists of a progressive loss of functional capacities with age, which has a strong impact on brain-related processes such as cognition or motor abilities [1]. The study of brain´s gray matter has drawn the attention of scientific community in the research of those aging-associated mechanisms underlying brain dysfunction and neurodegenerative disorders. However, the role of white matter (WM) is gaining momentum in this field [2].

The WM represents the portion of the brain composed of myelinated axons and myelin-producing glial cells, among others, and has an essential role in the efficient transmission of electric signals throughout the brain to enhance neuronal connectivity and a rapid information processing. With aging, the WM undergoes structural and functional alterations that can lead to neurological, behavioral and cognitive impairment, which may contribute to the cognitive decline and reduced neurogenesis reported in the elderly [2]. In addition, WM malfunction may influence the development and progression of neurodegenerative disorders such as Alzheimer´s disease (AD) and Parkinson´s disease [2, 3]. Among these age-related changes in WM, loss of volume and integrity, reduction of myelination, changes in the vasculature leading to hypoperfusion, and increased pro-inflammatory environment, have been reported [2].

The role of inflammation in aging and age-related disorders is still under debate, especially in the case of brain. However, it is clear that a chronic neuroinflammation occurs with age and that has a profound impact on normal brain function [4]. Concerning WM, a recent work reported that microglia-mediated inflammation is specifically increased with age and neurodegeneration (AD) in the WM of both humans and mice, with no additive effects, even in middle-age subjects [5]. In addition to microglia, other immune cells have been identified in human brain parenchyma with aging and age-related neurodegenerative diseases. Thus, CD3^+^ T cells and cytotoxic CD8^+^ T cells infiltrate the subventricular zone (SVZ) in aged individuals [6, 7], and also the AD brain [7–9].

Despite the increasing evidence that points out a relevant role of WM neuroinflammation in aging brain and neurodegeneration, there are scarce studies in humans that address this issue so far. In this work, we studied the presence of T cell infiltration and activated microglia in human white and gray matter from young and aged individuals, as well as in patients diagnosed with neurodegenerative disorders.

## RESULTS

### T cell infiltration is slightly higher in the white matter with physiological and pathological aging

**Figure 1:**
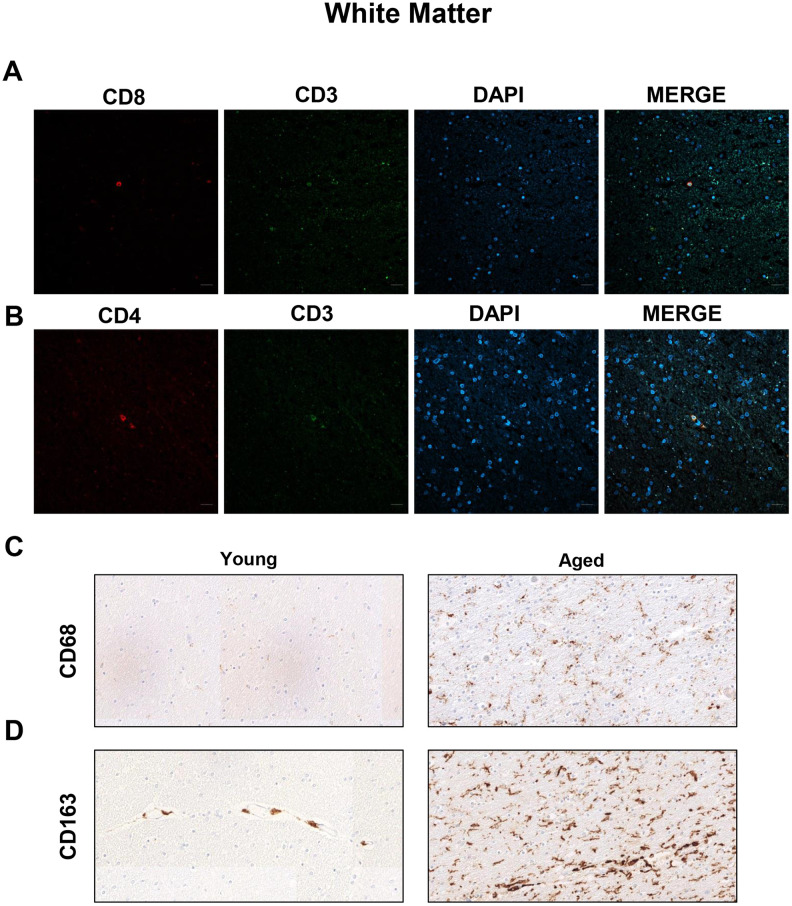
**Presence of cytotoxic CD8^+^ T cells in the white matter of aged individuals.** (**A**) Co-immunofluorescence of CD8 with CD3 marker (n=4). (**B**) Co-immunofluorescence of CD3 with CD4 marker (n=4). Cell nuclei were counterstained with DAPI. Scale bar: 20 μm. (**C**, **D**) Representative images of CD68 and CD163 microglia markers in young and aged individuals (n=3). Images appear to be spliced likely consequence of scanning and were obtained using the 20× objective.

To characterize the populations of T cells in the WM of aged individuals, we performed immunofluorescence analysis to detect CD8, CD4 and CD3 positive cells. Confocal analysis revealed that CD8^+^ cells were also positive for CD3 in aged individuals and patients with AD (Figure 1A and Supplementary Figure 1). There were, as well, cells co-stained for CD3 and CD4 markers in the same region (Figure 1B and Supplementary Figure 1). Additionally, WM from aged individuals presented enriched staining for CD68 and CD163, markers of microglia (Figure 1C, 1D). These results show the existence of cytotoxic CD8^+^ T cells in a pro-inflammatory environment in the WM of elderly individuals.

**Figure 2:**
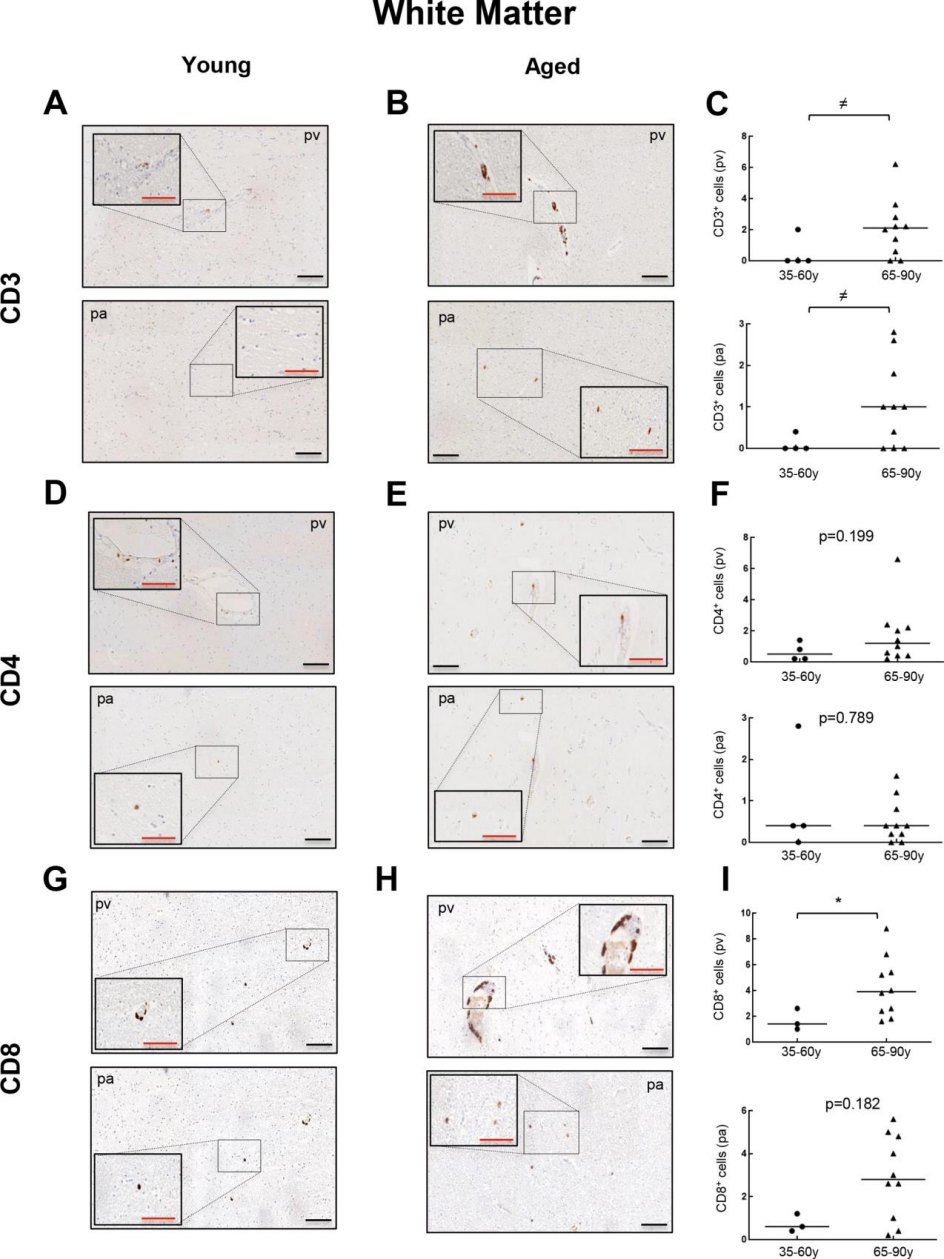
**Comparison of CD3^+^, CD4^+^ and CD8^+^ cells with physiological aging in white matter.** Representative images and quantification of CD3 (**A**–**C**), CD4 (**D**–**F**) and CD8 (**G**–**I**) expression in young and aged individuals. Data are presented as number of positive cells per mm^2^ with median bar. Red scale bar: 50 μm; black scale bar: 100 μm. White matter (WM), perivascular region (pv), brain parenchyma (pa).

Next, we performed immunohistochemistry (IHC) staining to evaluate the presence and distribution of CD3, CD4 and CD8 positive cells in the WM of young and old individuals. In the case of CD3^+^ cells, we found positive cells in 2 out of 4 young patients ranging from 0 to 2 cells per mm^2^ in perivascular region (pv; median of 0) and 0 to 0.4 cells per mm^2^ in brain parenchyma (pa; median of 0) (Figure 2A). In contrast, 8 out of 10 aged individuals presented CD3^+^ cells in WM, with 0 to 6.2 per mm^2^ in pv (median of 2.1) and 0 to 2.8 cells per mm^2^ in pa (median of 1) (Figure 2A–2C). With regard to CD4^+^ cells, they were found in all young and aged individuals analyzed but ranging from 0.2 to 1.4 cells per mm^2^ in pv (median of 0.5) and 0 to 2.8 cells per mm^2^ in pa (median of 0.4) in young individuals compared to 0.2 to 6.6 cells per mm^2^ in pv (median of 1.2) and 0 to 1.2 cells per mm^2^ in pa (median of 0.4) in the elderly group (Figure 2D–2F). Finally, CD8^+^ cells were also found in all cases from both groups: 1 to 2.6 cells per mm^2^ in pv (median of 1.4) and 0.4 to 1.2 cells per mm^2^ in pa (median of 0.6) in young individuals *versus* 1.6 to 8.8 cells per mm^2^ in pv (median of 3.9) and 0.2 to 5.6 cells per mm^2^ in pa (median of 2.8) in the aged ones (Figure 2G–2I). A homogeneous distribution for the three markers was noted in all groups, with no apparent regions that were particularly enriched in. Our data show that perivascular infiltration of positive cells for all markers occurs at higher levels than observed within WM parenchyma. Importantly, CD3^+^ and CD8^+^ cells are present at low levels but they are increased in aged individuals in both, perivascular and parenchyma, WM regions.

**Figure 3:**
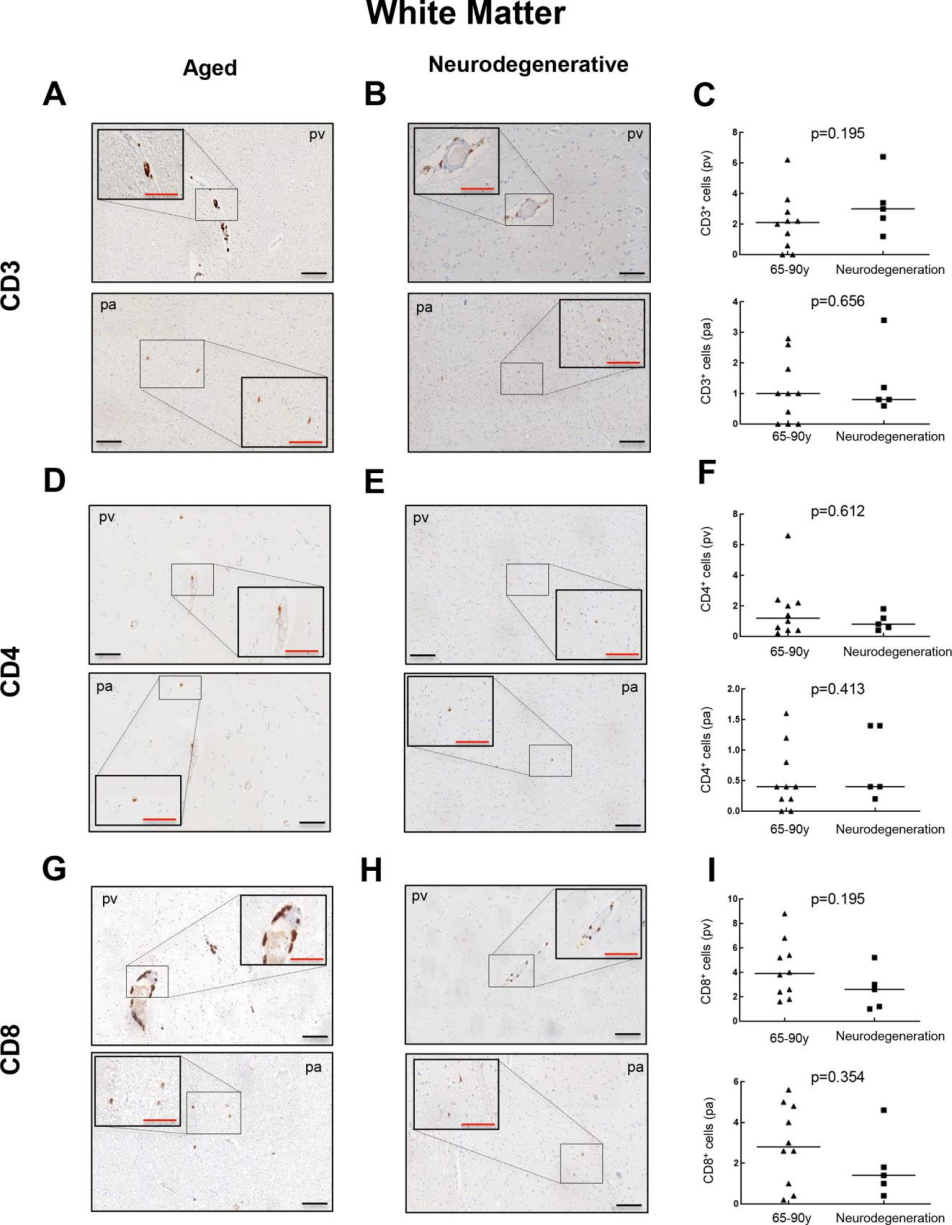
**Comparison of CD3^+^, CD4^+^ and CD8^+^ cells with pathological aging in white matter.** Representative images and quantification of CD3 (**A**–**C**), CD4 (**D**–**F**) and CD8 (**G**–**I**) expression in aged individuals and patients with neurodegenerative disease. Data are presented as number of positive cells per mm^2^ with median bar. Red scale bar: 50 μm; black scale bar: 100 μm. White matter (WM), perivascular region (pv), brain parenchyma (pa).

Then, we moved to patients diagnosed with a neurodegenerative condition (AD) to explore the presence of CD3^+^, CD4^+^ and CD8^+^ cells, and thus evaluate a potential impact of T cell infiltration in neurodegeneration. CD3^+^ cells were found in all 5 patients at similar levels to the aged group with 1.2 to 6.4 cells per mm^2^ in pv (median of 3) and 0.6 to 3.4 cells per mm^2^ in pa (median of 1) (Figure 3A–3C). For CD4^+^ cells, counts of 0.4 to 1.8 cells per mm^2^ in pv (median of 0.8) and 0.2 to 1.4 cells per mm^2^ in pa (median of 0.4) were obtained, maintaining again comparable levels to aged patients (Figure 3D–3F). Regarding CD8^+^ quantification, it ranged from 1 to 5.2 cells per mm^2^ in pv (median of 2.6) and 0.4 to 4.6 cells per mm^2^ in pa (median of 1.6) (Figure 3G–3I). Again, a homogeneous distribution of T cells throughout the regions studied was observed for all cases. These results indicate that T cell infiltration in the WM of neurodegenerative patients follows a similar trend to aged patients with no neuropathological signs.

### T cell infiltration also occurs in the enthorinal cortex of aged individuals with and without neurodegeneration

**Figure 4:**
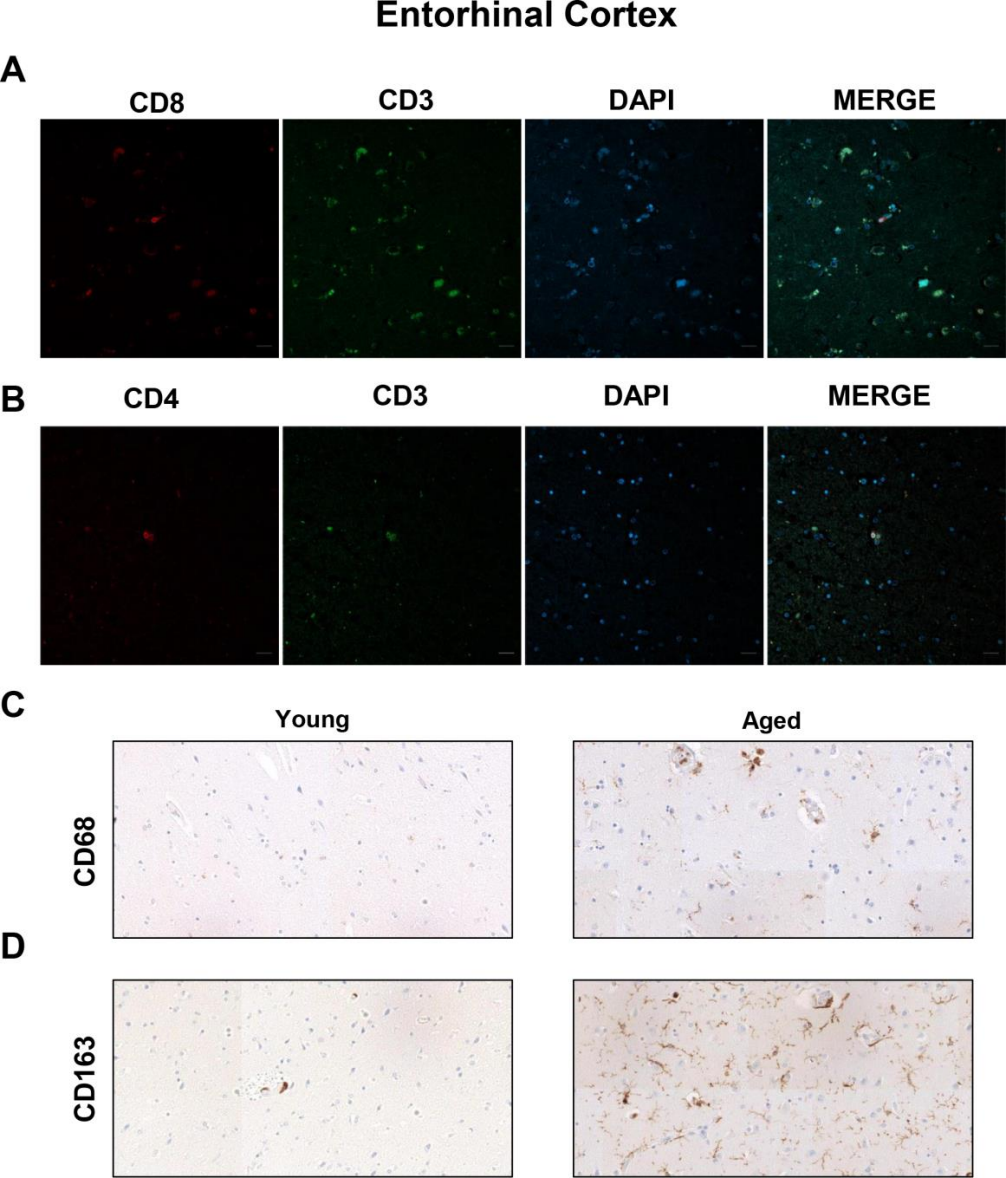
**Presence of cytotoxic CD8^+^ T cells in the entorhinal cortex of aged individuals.** (**A**) Co-immunofluorescence of CD8 with CD3 marker (n=4). (**B**) Co-immunofluorescence of CD3 with CD4 marker (n=4). Cell nuclei were counterstained with DAPI. Scale bar: 20 μm. (**C**, **D**) Representative images of CD68 and CD163 microglia markers in young and aged individuals (n=3). Images were obtained using the 20× objective.

**Figure 5:**
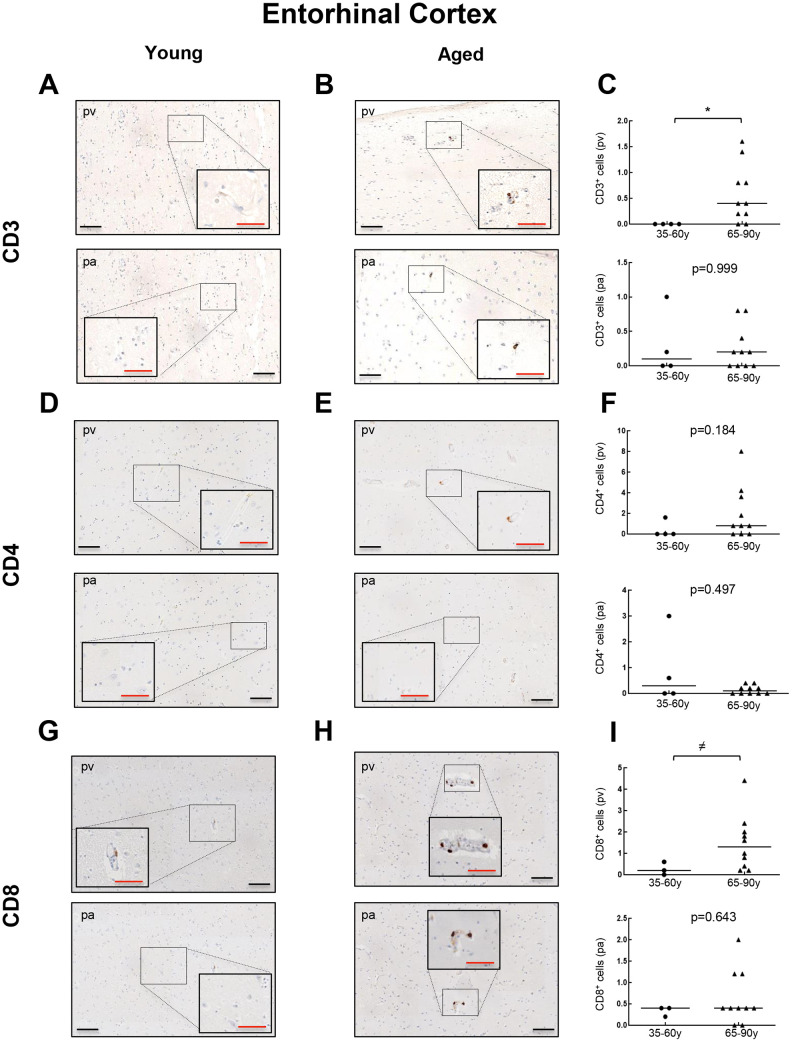
**Comparison of CD3^+^, CD4^+^ and CD8^+^ cells with physiological aging in entorhinal cortex.** Representative images and quantification of CD3 (**A**–**C**), CD4 (**D**–**F**) and CD8 (**G**–**I**) expression in young and aged individuals. Data are presented as number of positive cells per mm^2^ with median bar. Red scale bar: 50 μm; black scale bar: 100 μm. Entorhinal cortex (EC), perivascular region (pv), brain parenchyma (pa).

**Figure 6:**
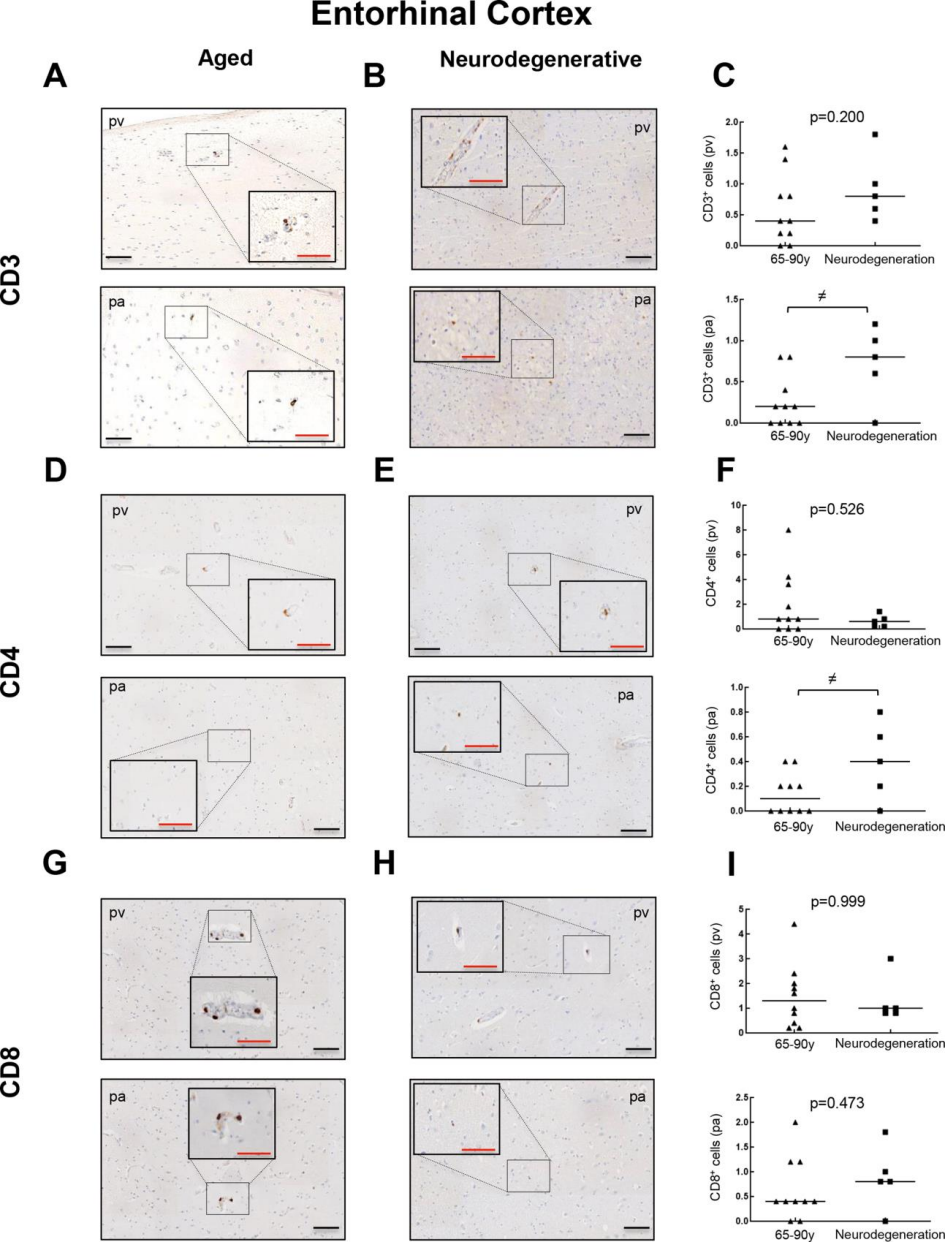
**Comparison of CD3^+^, CD4^+^ and CD8^+^ cells with pathological aging in entorhinal cortex.** Representative images and quantification of CD3 (**A**–**C**), CD4 (**D**–**F**) and CD8 (**G**–**I**) expression in aged individuals and patients with neurodegenerative disease. Data are presented as number of positive cells per mm^2^ with median bar. Red scale bar: 50 μm; black scale bar: 100 μm. Entorhinal cortex (EC), perivascular region (pv), brain parenchyma (pa).

To further explore the presence of T cell infiltration in the brain, we moved to the enthorinal cortex (EC) and quantified those CD3, CD4 and CD8 positive cells in our patients. As in the case of WM, we detected the co-expression of CD8^+^-CD3^+^ and CD3^+^-CD4^+^ cells in elderly individuals and patients with AD (Figure 4A, 4B and Supplementary Figure 2). Similarly, CD68 and CD163 markers were elevated in the elderly compared to young individuals (Figure 4C, 4D). Next, we observed that cell counts for the three markers were less than 1 cell per mm^2^ in all young patients except one who presented herpes viremia and severe liver failure. In contrast, we obtained higher values for aged patients as follows: 7 out of 10 individuals showed CD3^+^ cell infiltration with 0 to 1.6 cells per mm^2^ in pv (median of 0.4) and 0 to 0.8 cells per mm^2^ in pa (median of 0.2) (Figure 5A–5C). In the case of CD4^+^ cells, aged individuals had 0 to 8 cells per mm^2^ in pv (median of 0.8) and 0 to 0.4 cells per mm^2^ in pa (median of 0.1) (Figure 5D–5F). Finally, we found levels of 0.2 to 4.4 cells per mm^2^ in pv (median of 1.3) and 0 to 2 cells per mm^2^ in pa (median of 0.4) for CD8^+^ cells (Figure 5G–5I). Similar values were obtained from patients with neurodegeneration, ranging from 0.4 to 1.8 cells per mm^2^ in pv (median of 0.8) and 0 to 1.2 cells per mm^2^ in pa (median of 0.7) for CD3^+^ cells (Figure 6A–6C), 0.2 to 1.4 cells per mm^2^ in pv (median of 0.6) and 0 to 0.8 cells per mm^2^ in pa (median of 0.5) for CD4^+^ cells (Figure 6D–6F), and 0.8 to 3 cells per mm^2^ in pv (median of 1) and 0 to 1.8 cells per mm^2^ in pa (median of 0.9) for CD8^+^ cells (Figure 6G–6I). Overall, these results reveal that T cell infiltration in EC is lower than that found in the WM of the same individuals. Moreover, the presence of infiltrating T cells in EC is also higher with physiological aging and neurodegeneration, maintaining similar levels between these two groups of patients.

## DISCUSSION

T cell infiltration in human brain parenchyma with normal aging remains a largely unexplored field, despite the well-known development of a chronic, pro-inflammatory environment in the aged brain that impact its normal function [10]. These data are especially scarce in WM, to which little attention has been paid. Trying to provide new insight in this field, we report a low-grade infiltration of CD3^+^ and CD8^+^ cells in the WM and EC of aged individuals, also with neurodegenerative disorders. Intriguingly, we detected a higher proportion of CD8^+^ cells than CD3^+^ cells, which might suggest some methodological limitations or the presence of at least one CD8^+^ cell subpopulation different from cytotoxic CD3^+^/CD8^+^ T cells. In this sense, some natural killer cells carry CD8 but not CD3 [11]. Despite the low levels, we show an increase of CD3^+^ and CD8^+^ cells in the WM of aged individuals and neurodegenerative patients compared to young ones, in both perivascular and parenchymal regions. Conversely, lower level of T infiltration is observed in the EC, where less than 2 cells per mm^2^ for all markers were found to infiltrate the parenchyma in the vast majority of patients. The number of positive cells is lower than the increase of CD3^+^ cells, and especially CD8^+^ cells, in the SVZ of old individuals [6, 7], and in those individuals who displayed neurodegeneration in our cohort and others [7, 8]. However, T cell infiltration seems to be widespread in the WM of aged mice, where CD3^+^ T cells are predominantly accumulated with aging [12]. In this sense, infiltration of CD3^+^, CD4^+^ and CD8^+^ cells into the brain parenchyma and perivascular regions is also more frequent in aged mice compared to young ones after cytokine stimulation [13]. It is therefore tempting to suggest that T cell infiltration in humans with aging is mostly associated with stem regions such as SVZ, where it would have a stronger impact mainly through the cytotoxic activity of CD8^+^ cells, rather than EC or WM. We also report an increased expression of CD68 and CD163 microglia markers, which suggests a pro-inflammatory environment in the aged WM and cortex. Elevation of microglia, measured by CD68 and IBA1, was also observed in the SVZ neurogenic niche of the same cohort used in this study [7]. It does seem that neuroinflammation mediated by microglia activation is predominant in the WM with aging and neurodegeneration [5], which could indirectly reinforce a role of T cell infiltration in the SVZ with physiological and pathological aging.

Interestingly, we found an increase for CD3^+^ and CD8^+^ cells in neurodegenerative patients (all of them with diagnosis of AD) compared to young ones in all regions analyzed excluding EC parenchyma, which is in line with our results in aged patients without neuropathological lesions. Additional works have reported a marked increment of CD8^+^ cells in AD brains compared to healthy aged brains [8, 14, 15], including hippocampal and cortical regions. Although differences in antibodies, IHC protocols, the anatomical regions explored and also different criteria for T cell quantification (*e.g.*, we excluded those T cells inside blood vessels) might explain these controversial results, our data highlight that T cell infiltration seems not to be elevated in AD pathogenesis from a previous non-neuropathological state, but also it might be contributing to a yet established pathological context.

In summary, we report a low degree of T cell infiltration in human white and gray matter, at similar levels in both aged individuals with no neuropathological injuries and neurodegeneration, being especially remarkable in the case of WM. T cell infiltration also correlates with the presence of a pro-inflammatory state. These results reinforce the role of T cells in brain during physiological and pathological aging, but also point out a more relevant role of T cell-related inflammation in critical areas for neurogenesis such as the SVZ rather than WM or cortical regions such as the EC.

## MATERIALS AND METHODS

### Samples

Human brain samples (n=19) were collected from autopsies conducted at Donostia University Hospital (Spain). Postmortem interval (PMI) was limited to 12 hours due to its effects on brain proteins. Brains were kept in a fixative solution (4% paraformaldehyde) for a period of not less than 24 h. Samples were divided in three groups: “young” (n=4; individuals ranged from 36 to 58 years old), “old” (n=10; individuals aged 65-87 years old) and “neurodegenerative” (n=5; individuals between 60-90 years old). Inclusion criteria for selection of “young” and “old” patients included the absence of diagnosis of neurodegenerative disorders as well as the lack of neuropathological injuries in the regions analyzed. Patient´s information is described in Table 1. This study was approved by the Clinical Research Ethics Committee of the Donostia University Hospital (AMM-MHP-2019-1) and adhered to the tenets of the Declaration of Helsinki by the World Medical Association regarding human experimentation.

**Table 1:** Clinical information of individuals included in this study.

**Group**	**Age**	**Gender**	**Diagnosis**
Young	43	M	Severe acute liver failure and herpes viremia
	36	F	Toxic shock
	47	M	Neuroendocrine carcinoma and encephalopaty
	58	M	Disseminated signet ring cell carcinoma
Old	66	M	Disseminated breast carcinoma
	67	M	Laryngeal squamous cell carcinoma and respiratory depression
	65	M	Pulmonary hemorrhage
	77	M	Septic shock
	74	F	Disseminated breast carcinoma
	85	M	Malignat mesothelioma
	78	F	Hypovolemic shock
	70	F	Metastatic melanoma
	85	M	Subdural hematoma and sudden death
	87	M	Empyema
Neurodegenerative	90	F	Alzheimer´s disease
	79	M	Alzheimer´s disease
	71	M	Alzheimer´s disease
	60	F	Alzheimer´s disease
	65	F	Alzheimer´s disease

### Immunohistochemistry of brain sections

IHC was performed following standard procedures. Briefly, whole brains were extracted, fixed in formalin, paraffin-embedded and subsequently sectioned in 5 μm coronal sections with a Leica rotary microtome. Sections were taken at lateral geniculate nucleus of the thalamus in the temporal lobe, which was considered our topographic reference point. All sections included hippocampus, temporal horn and EC. The following primary antibodies were used: CD3 (Roche, Ref.: 790-4341, Clone: 2GV6), CD4 (Roche, Ref.: 790-4423, Clone: SP35), CD8 (Roche, Ref.:790-4460, Clone: SP57), CD68 (Roche, Ref.: 790-2931, Clone: KP-1) and CD163 (Roche, Ref: 760-4437, Clone: MRQ-26). IHC was performed following the manufacturer´s instructions on the Roche Ventana Benchmark ULTRA System with ethylenediaminetetraacetic acid (EDTA) pH 8.5 antigen retrieval. Sections were visualized with a light microscope using the 10×, 20× and 40× objective and then scanned with Virtuoso v.5.6.1 software (Ventana Medical Systems, Roche).

### Immunofluorescence of brain sections

Immunofluorescence was performed in formalin fixed brain samples. Paraffin embedded tissue sections were deparaffinized in xylene and rehydrated in a series of graded alcohols and then heated in citrate buffer for 30 min for antigen retrieval. Tissues were permeabilized with 0.5% Triton X-100 (PBS-T) and blocked for 1 h with 1% bovine serum albumin and 5% goat-serum in PBS-T. Sections were incubated at 4ºC overnight with the following primary antibodies: anti-CD3 (ab5690, Abcam), CD4 (ab133616, Abcam) and CD8 (ab4055, Abcam). The sections were washed 3 times for 5 min with PBS 0.1% tween-20 and incubated for 1 h at room temperature in darkness with Alexa Fluor 488 goat anti-rabbit (Invitrogen) and Alexa Fluor 555 goat anti-rabbit (Invitrogen) secondary antibodies. Nuclear DNA was stained with DAPI (Sigma-Aldrich). The preparation was mounted with Fluoro-Gel mounting media and immunofluorescence was evaluated with the Zeiss LSM 900 confocal microscope.

### T cell quantification

The quantification of T cells positive for the different markers was manually performed in entire coronal sections from previously scanned images. Two brain regions were checked: EC and the WM associated. We randomly selected five areas per region and defined a visual field of 1 mm^2^ each, within which all T cells were considered. We counted all parenchyma-infiltrating positive cells (pa) and also those located in the vascular walls (pv). T cells were identified as CD3^+^ cells, helper T cells as CD4^+^ and cytotoxic T cells as CD8^+^.

### Statistical analysis

The number of T cells was expressed as average number of positive cells per unit area (mm^2^) for each marker. Two-tailed Mann-Whitney U test was performed to compare CD3, CD4 and CD8 positive cell counts between groups. Asterisks (≠, *, ** and ***) indicate statistically significant differences (p<0.1, p<0.05, p<0.01 and p<0.001, respectively).

## Supplementary Material

Supplementary Figures
