# Supplementary material for: CD8+ T cells are present at low levels in the white matter with physiological and pathological aging
Source: Aging (Albany NY). 2020 Oct 13;12(19):18928–41. doi: 10.18632/aging.104043 (PMC7732290; doi:10.18632/aging.104043)
Supplement: Supplementary Figures [file aging-12-104043-s001..pdf]

SUPPLEMENTARY FIGURES

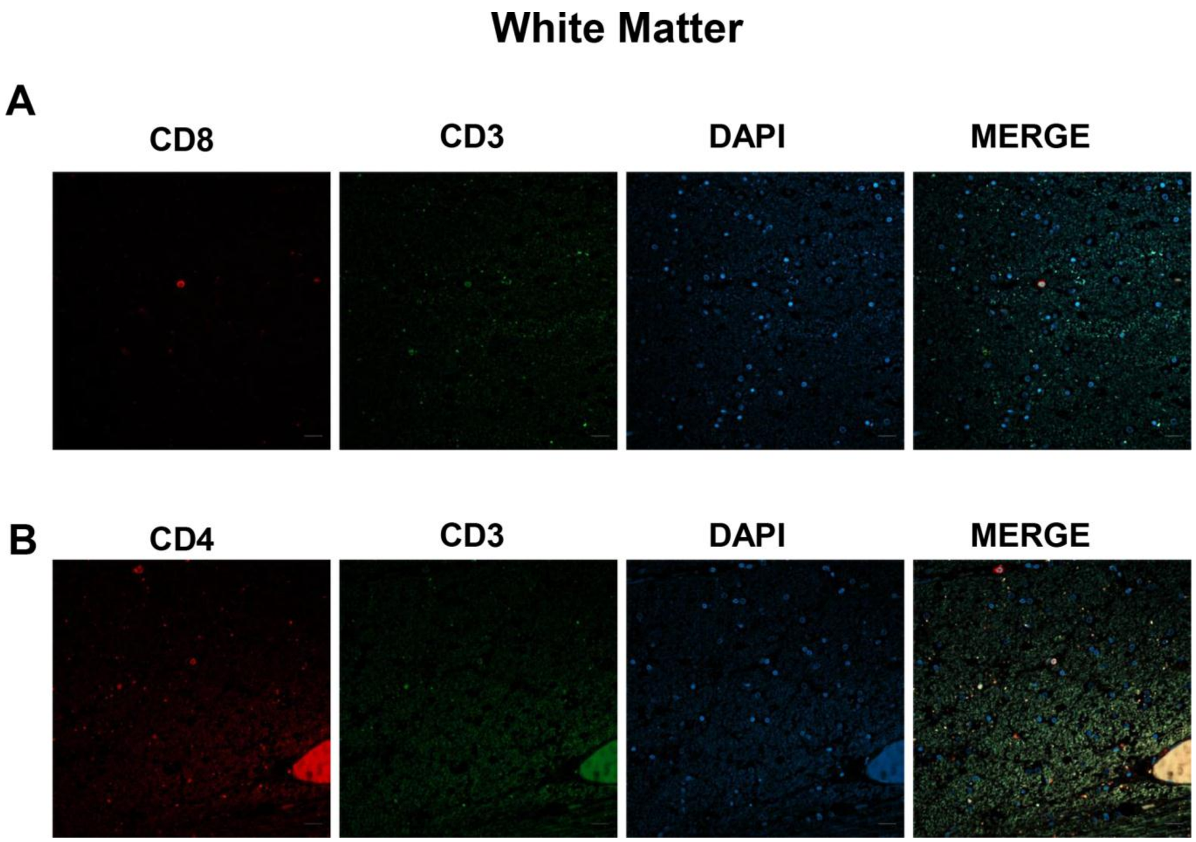

Supplementary Figure 1. Presence of cytotoxic CD8$^+$ cells in white matter of patients with neurodegenerative diseases. (**A**) Co-immunofluorescence of CD8 with CD3 marker. (**B**) Co-immunofluorescence of CD3 with CD4 marker (n=2). Cell nuclei were counterstained with DAPI. Scale bar: 20 μm.

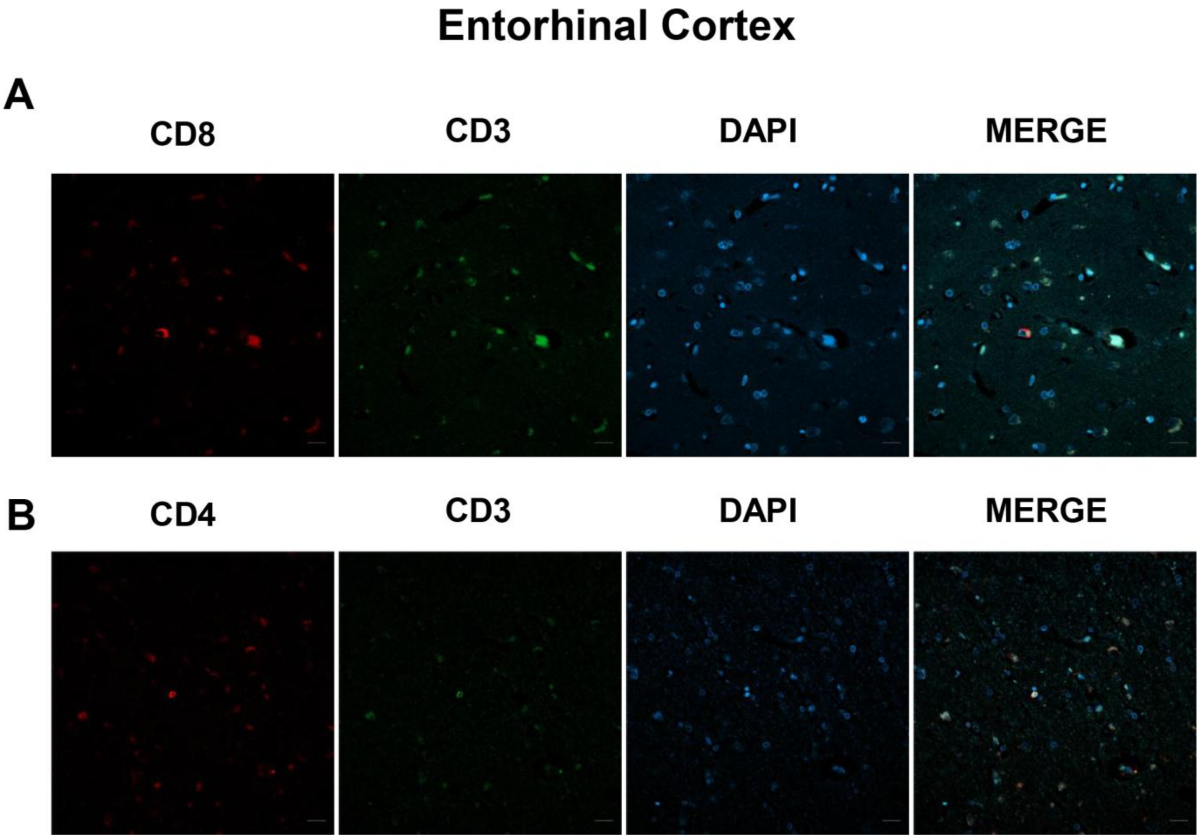

Supplementary Figure 2. Presence of cytotoxic CD8^{+} cells in entorhinal cortex of patients with neurodegenerative diseases. (**A**) Co-immunofluorescence of CD8 with CD3 marker. (**B**) Co-immunofluorescence of CD3 with CD4 marker (n=2). Cell nuclei were counterstained with DAPI. Scale bar: 20 µm.
